# B-cell epitope mapping of VP5 protein of bluetongue virus serotype 1 using monoclonal antibodies

**DOI:** 10.1016/j.virusres.2025.199663

**Published:** 2025-11-09

**Authors:** Fanhua Meng, Xuechun Liu, Yuqing Song, Xinbing Hu, Zhancheng Tian, Guiquan Guan, Lijie Tang, Hong Yin, Junzheng Du

**Affiliations:** a State Key Laboratory for Animal Disease Control and Prevention, College of Veterinary Medicine, Lanzhou University, Lanzhou Veterinary Research Institute, Chinese, Academy of Agricultural Sciences, Lanzhou 730000, China; b Gansu Province Research Center for Basic Disciplines of Pathogen Biology, Lanzhou 730046, China; c College of Veterinary Medicine, Northeast Agricultural University, Harbin 150030, China; d Jiangsu Co-innovation Center for Prevention and Control of Important Animal Infectious Diseases and Zoonoses, Yangzhou University, Yangzhou 225009, China

**Keywords:** B-cell epitope, Bluetongue virus serotype 1, VP5Δ1–79aa protein, Monoclonal antibody

## Abstract

Bluetongue (BT) is a severe infectious disease affecting ruminants, caused by the bluetongue virus (BTV), an *Orbivirus* transmitted by *Culicoides* midges. VP5 is a major component of the outer capsid of BTV, and a key constituent of BTV subunit and virus-like particle vaccines. However, to date, the B-cell epitopes on VP5 recognized by humoral immune responses remain unidentified. In this study, recombinant VP5Δ1–79aa was expressed in *Escherichia coli* (*E. coli*) and purified for mouse immunization. Five monoclonal antibodies (mAbs) were identified through hybridoma fusion, clonal selection, and immunological assays. B-cell epitopes were recognized by mAbs 4G9, 6C9, 3A1, 3C8 and 6B1, these epitopes were mapped using a series of truncated overlapping peptides expressed as glutathione S-transferase fusion proteins. 4G9 recognized the ^144^DEKQFDILNK^153^ sequence, 6C9 recognized ^154^AVTSYNKILT^163^, 3A1 and 3C8 recognized ^207^VERDGMQEEA^216^, and 6B1 recognized ^312^ENHKELMHIK^321^. These specific mAbs and their corresponding B-cell epitopes provide valuable insights into the structure and function of VP5, contributing to the advancement of serological diagnostic methods and development of epitope-based vaccines for BTV.

## Highlights

• Truncation of the 79 N-terminal amino acids significantly facilitated VP5 expression in *Escherichia coli*, enabling the production of recombinant VP5Δ1–79aa.
• Five monoclonal antibodies (mAbs) specific to BTV1 VP5 were generated using hybridoma technology.
• Comprehensive epitope mapping with the generated mAbs identified four novel linear B-cell epitopes within the BTV-1 VP5 protein.
• These findings provide critical insights for the development of diagnostic tools and vaccines against BTV-1.

## 1 Introduction

Bluetongue virus (BTV) is the etiological agent of bluetongue (BT), which primarily affects domestic and wild ruminants (Wilson and Mellor, 2009). Clinical manifestations include high fever, tachypnea, and ulcerative lesions, which can progress to death in severe cases (Maclachlan et al., 2009). To date, 36 BTV serotypes have been described (Ries et al., 2021). For many years, the geographical range of BTV was believed to be circumscribed by the latitudinal boundaries of 40°–50°N and 20°–30°S, making it primarily endemic to tropical and subtropical regions (Carpenter et al., 2015; Hudson et al., 2023; MacLachlan and Osburn, 2006). However, since the late 20th century, multiple BT outbreaks in Europe have been linked to strains believed to spread from North Africa into Southern Europe (Kundlacz et al., 2019).

BTV belongs to the genus *Orbivirus* within the family *Sedoreoviridae*. Its genome consists of 10 segments of double-stranded RNA (S1–S10), encoding seven structural proteins (VP1–7) and five non-structural proteins (NS1–5) (Belhouchet et al., 2011; Forzan et al., 2004; Ratinier et al., 2011; Roy, 2005). VP1 (RNA-dependent RNA polymerase), VP4 (capping enzyme), and VP6 (RNA helicase) constitute the transcriptional complex (Boyce et al., 2004; Grimes et al., 1998; Stäuber et al., 1997; Sutton et al., 2007). VP3 and VP7 form the inner capsid, which houses the transcriptional complexes and double-stranded RNA fragments, known as the core particle (Patel and Roy, 2014). VP5 and VP2 form the outer capsid, which is added to the periphery of the core particle and is essential for the maturation of BTV particles (Bhattacharya and Roy, 2008).

VP5 is the second most variable structural protein of BTV, with 41–79 % amino acid identity among different serotypes; second only to VP2 in sequence diversity (Maan et al., 2011). Two capsid proteins, VP2 and VP5, are responsible for entry into the host cytosol (Hassan et al., 2001; Hassan and Roy, 1999). VP2 (110 kDa) binds to the cell surface and facilitates receptor-mediated endocytosis of the viral particles. VP5 (60 kDa) is capable of penetrating the host cell membrane and delivering 75-nm core particles into the cytosol (Forzan et al., 2007; Patel et al., 2016). VP5 promotes escape of the virus from late endosomes by sensing low pH and participating in its membrane penetration (Forzan et al., 2004). Identification of the specific antigenic epitopes recognized by host antibodies is crucial for understanding the natural immune response to infection, as well as developing epitope-based marker vaccines and diagnostic tools (Sukupolvi-Petty et al., 2010). Many vaccines are designed to induce humoral immune responses that target specific epitopes present on the surface proteins of pathogens, thereby providing protection against future exposure to the pathogen (Xu and Kulp, 2019).

In this study, VP5Δ1–79aa was expressed in *E. coli*. Five mAbs against VP5Δ1–79aa were generated, and four novel epitopes were accurately identified. Cross-reactivity analysis indicated that mAbs3A1, 4G9, 6C9, 3C8, and 6B1 specifically recognized the VP5 protein of BTV-1. This study provides valuable insights for further investigation into the biological functions of VP5, laying the foundation for the development of serological diagnostic assays for BTV and the design of epitope-based peptide vaccines as well as subunit vaccines.

## 2 Materials and methods

### 2.1 Cells, animals, plasmids and serum

Mouse myeloma cells (SP2/0) were cultured in RPMI 1640 medium (Gibco, USA), supplemented with 20 % fetal bovine serum (FBS; Gibco, USA). BHK-21 cells were cultured in a 37 °C incubator with 5 % CO_2_, using Dulbecco's modified Eagle's medium supplemented with 5 % FBS (Gibco, USA). The BTV-1 strain (GS/11), anti-BTV-1 rabbit positive serum, anti-6 ×  His tag mAb and recombinant VP5 proteins of African horse sickness virus serotype 1 (AHSV-1) and EHDV-1, were preserved in the laboratory.

### 2.2 Expression and identification of recombinant VP5Δ1–79aa

Based on the amino acid sequence of the VP5 protein from the reference strain of BTV serotype 1 (GenBank accession: ACR58462.1), its hydrophobicity was analyzed using DNAStar software. The N-terminal region was removed due to the cytotoxicity of its VP5 dagger domain, which severely limits protein expression (Hassan et al., 2001). This conclusion is further bolstered by epitope prediction results, which indicate poor antigenicity that largely rules out the presence of relevant epitopes in this segment. After truncating the nucleotide sequence encoding the N-terminal 79 amino acids, the VP5Δ1–79aa gene was optimized according to the codon preferences for *E. coli* expression. The VP5Δ1–79aa gene was synthesized by GeneCreate Biological Engineering Co. Ltd. (Wuhan, China) and ligated into the expression vector pET-28a-SUMO. The resulting recombinant plasmid was designated pET-sumo-VP5Δ1–79aa and transformed into *E. coli* BL21 (DE3) competent cells (TransGen Biotech, Beijing, China). Protein expression was induced by adding 1 mM isopropyl β-d-1-thiogalactopyranoside (IPTG) at 16 °C. After IPTG induction, the expression products were collected at intervals of 2 h after IPTG induction for analysis. The precipitate obtained after ultrasonic lysis was purified at 16 h. The inclusion bodies were solubilized using a prepared solution (100 mM NaH_2_PO_4_, 10 mM Tris–HCl, and 8 M urea) according to the manufacturer's instructions for Ni-NTA agarose (Qiagen, Germany).The purified recombinant VP5Δ1–79aa protein was renatured in urea solutions with varying concentrations and identified using anti-6 ×  His tag monoclonal antibody (Proteintech, Wuhan, China,)and anti-BTV-1 rabbit positive serum.

### 2.3 Preparation of VP5Δ1–79aa mAbs

Six-week-old female BALB/c mice were subcutaneously immunized with a mixture containing 50 µg VP5Δ1–79aa and an equal volume of complete Freund's adjuvant (Sigma, USA). A booster injection, comprising VP5Δ1–79aa and incomplete Freund's adjuvant, was administered every 2 weeks. At 14 days following the third injection, the serum titer was determined by indirect ELISA. Mice with the highest titer were immunized with VP5Δ1–79aa. Three days later, spleen cells were fused with SP2/0 cells using polyethylene glycol (PEG; Sigma, USA). The fused cells were screened in 96-well plates using HAT (Sigma, USA) selection medium. After 7 days, the hybridomas were cultured in HT (Sigma, USA) medium. When hybridoma cell growth covered more than one-third of the well surface area, the supernatants were analyzed by indirect ELISA. Positive hybridoma cells were subcloned 3–5 times using the limited dilution method. The specificity and reactivity of the antibodies were assessed using western blotting and immunofluorescence assay.

### 2.4 ELISA

VP5Δ1–79aa was diluted to 0. 5μg/mL with sodium bicarbonate buffer (pH 9.6), coated onto a 96-well microplate, and incubated overnight at 4 °C. After washing the wells three times with phosphate-buffered saline containing 0.05 % Tween-20 (PBST), a blocking solution containing 5 % skimmed milk powder was added, and the plate was incubated at 37 °C for 1 h. Following another three washes with PBST, 100 μL hybridoma supernatant was added to each well, and the plate was incubated at 37 °C for 1 h. After a subsequent series of washes, a 1:10,000 dilution of horseradish-peroxidase-conjugated goat anti-mouse IgG (Abcam, UK) was added at 100 μL per well and incubated at 37 °C for 1 h. Following three additional washes with PBST, 100 μL chromogenic substrate solution (TMB) was added and incubated for 10 min. The reaction was terminated with 0.5 M sulfuric acid, and the optical density was measured at 450 nm using the Multiskan Sky High (Thermo, USA) full-wavelength microplate reader.

### 2.5 Western blotting

The reactivity of the mAbs with VP5 was analyzed in BHK-21 cells following 12 h infection with BTV-1 at an MOI of 1. The protein lysate from BTV-1-infected BHK-21 cells was separated by SDS-PAGE and subsequently transferred to a polyvinylidene fluoride (PVDF) membrane. The PVDF membranes were blocked with 5 % skimmed milk in PBST at 37 °C for 1 h. The membranes were incubated with hybridoma supernatants in a blocking buffer at 4 °C overnight. After three washes with PBST, the PVDF membranes were incubated with horseradish-peroxidase-conjugated goat anti-mouse IgG (1:10,000; Abcam, UK) at 37 °C for 1 h. Following three additional washes with PBST, enhanced chemiluminescence substrate (Abbkine, Wuhan, China) was added to the PVDF membranes, and the protein signal was visualized and captured using a ChemiDoc XRS+ Imaging System (Bio-Rad, USA).

### 2.6 Immunofluorescence assay

After infection with BTV-1 at an MOI of 1 for 12 h, the cells were fixed at room temperature with precooled 4 % paraformaldehyde for 10 min and permeabilized with 0.1 % Triton X-100 in PBS for 10 min. The cells were blocked with 3 % bovine serum albumin for 1 h. The cells were incubated with hybridoma supernatant as primary antibodies for 1 h at room temperature. After washing three times with PBS, the cells were incubated with Alexa-Fluor-594-conjugated goat anti-mouse IgG, diluted to 2 μg/mL (Abcam), for 1 h. The cell nuclei were stained with 1μg/mL Hoechst 33,342 solution (Thermo, USA) for 10 min. Finally, the cells were treated with Anti-Fade Mounting Medium (Beyotime, Shanghai, China) and observed under a Zeiss LSM980 (Carl Zeiss AG, Germany) scanning confocal microscope.

### 2.7 B-cell epitope mapping

**Fig. 1:**
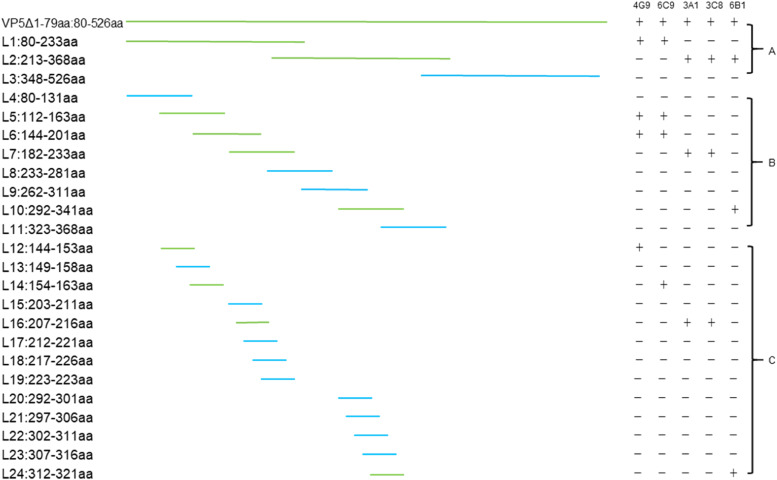
Schematic diagram of the VP5Δ1–79aa truncation localization strategy. The fragments recognized by mAbs 4G9, 6C9, 3A1, 3C8, and 6B1 are indicated by green lines, while the fragments not recognized mAbs are represented by blue lines. (A), (B), and (C) denote the three rounds of truncation.

To identify the B-cell epitopes recognized by mAbs on VP5Δ1–79aa, a series of truncated recombinant VP5Δ1–79aa fragments were expressed and characterized. Three overlapping truncated VP5 fragments were designed for the first round of antigenic peptide mapping, while 20 overlapping amino acid residues were selected for the second and third rounds of peptide localization (Fig.1). These truncated gene fragments were synthesized and cloned into the pGEX-6p-1 expression vector by GeneCreate Biological Engineering Co. Ltd (Wuhan, China). The glutathione S-transferase-tagged (GST) recombinant proteins were expressed in *E. coli* BL21(DE3) and verified by western blotting. The expressed proteins were used to map the B-cell epitopes recognized by the mAbs.

### 2.8 Bioinformatic analysis of epitopes

To verify the conservation of the identified VP5Δ1–79aa epitopes, the VP5Δ1–79aa sequences of three *Orbiviruses* BTV, AHSV, and EHDV were retrieved from GenBank. Amino acid sequence alignment and conservation analysis were performed using BioEdit software. VP5 is the membrane fusion and penetration protein of BTV and consists of three domains: dagger (M1-S68), unfurling (K69-F354), and anchoring (I355-A526) (Zhang et al., 2016). The spatial distribution and structure of the VP5 (PDB: 3J9E) epitope were visualized and analyzed using PyMOL. Cross-reactivity of mAbs with recombinant AHSV VP5 and EHDV VP5 proteins was analyzed by western blotting.

## 3 Results

### 3.1 Expression and purification of recombinant VP5Δ1–79aa protein

**Fig. 2:**
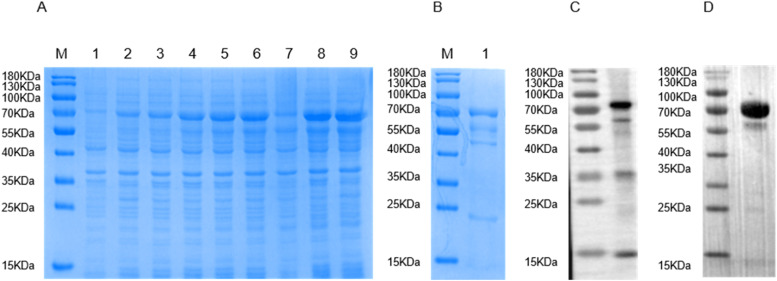
Expression, purification, and identification of VP5Δ1–79aa protein. (A) SDS-PAGE identification of the expression of VP5Δ1–79aa. Lane 1: uninduced bacteria; lanes 2–9: bacteria induced for 2, 4, 6, 8, 10, 12, 14 and 16 h. (B) Purified VP5Δ1–79aa verified by SDS-PAGE. Recombinant VP5Δ1–79aa was identified by (C) anti-6 ×  His tag mAb and (D) BTV-1 positive serum.

The recombinant plasmid pET-sumo-VP5Δ1–79aa was transformed into BL21 (DE3) competent cells for protein expression. The bands resulting from SDS-PAGE showed that the VP5Δ1–79aa protein had a molecular weight of 70 KDa (Fig. 2A). The VP5Δ1–79aa was purified from the precipitate by Ni–NTA Agarose and the results were confirmed by SDS-PAGE (Fig. 2B). Western blotting verified that the purified VP5Δ1–79aa reacted with the anti-6 ×  His tag mAb (Fig. 2C) and anti-BTV-1 rabbit positive serum (Fig. 2D).

### 3.2 Preparation and identification of VP5Δ1–79aa monoclonal antibody

**Fig. 3:**
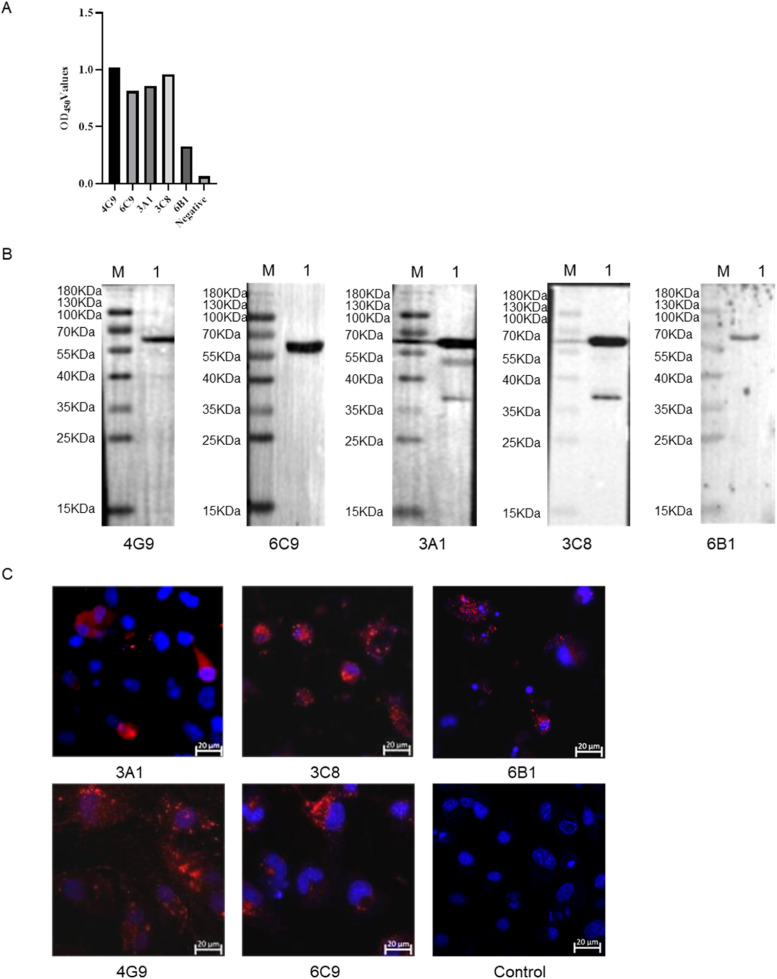
Identification of mAbs. (A) Reactivity of mAbs analyzed by indirect ELISA. (B) Reactivity of mAbs analyzed by western blotting. Lane 1: BTV-1-infected BHK-21 cells. (C) Immunofluorescence assay of BTV-1-infected BHK-21 cells using the mAbs. Scale bar = 20 μm.

After three rounds of screening and subcloning, five hybridoma cell lines (4G9, 6C9, 3A1, 3C8, and 6B1) that stably secreted anti-VP5Δ1–79aa mAbs were obtained through limited dilution and indirect ELISA (Fig. 3A). The specific reactivity of mAbs 4G9, 6C9, 3A1, 3C8, and 6B1 with VP5 in BTV-1-infected BHK-21 cells was confirmed by western blotting (Fig. 3B) and immunofluorescence assays (Fig. 3C).

### 3.3 Identification of antigenic epitopes by mAbs

**Fig. 4:**
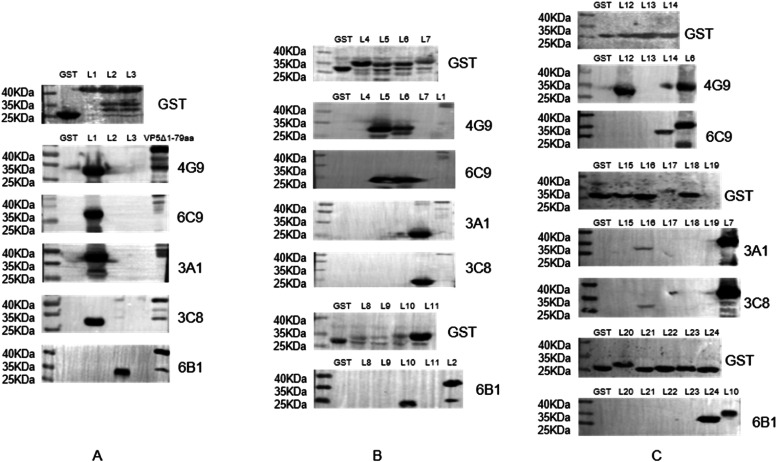
Precise localization of antigenic epitopes using mAbs. The primary (A), secondary (B), and tertiary (C) VP5Δ1–79aa fusion proteins with truncated prokaryotic expression were screened. The antigenic epitopes of the five mAbs were displayed by western blotting. The empty vector pGEX-6p-1-GST and the VP5Δ1–79aa were used as negative and positive controls, respectively.

**Fig. 5:**
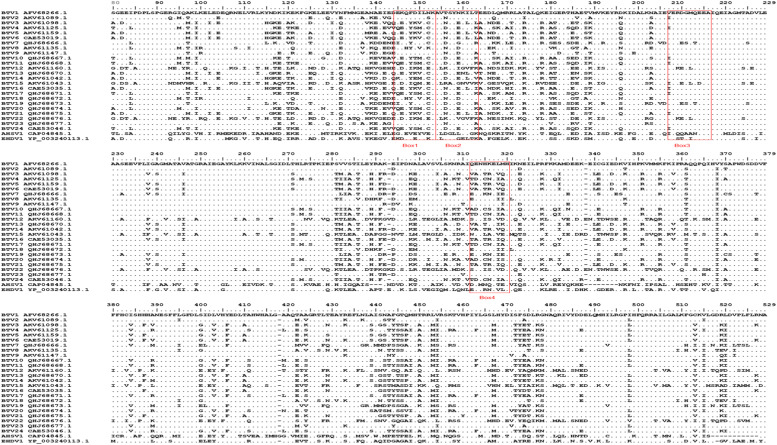
Sequence alignment of VP5 from three Orbiviruses: ^144^DEKQFDILNK^153^ (Box 1) identified by 4G9; ^154^AVTSYNKILT^163^ (Box 2) identified by 6C9; ^207^VERDGMQEEA^216^ (Box 3) identified by 3A1 and 3C8; and ^312^ENHKELMHIK^321^ (Box 4) identified by 6B1 are circled in red.

The overlapping truncated VP5Δ1–79aa fragment with a GST tag was expressed in *E. coli*, and western blotting was performed to determine the antigenic epitopes. VP5Δ1–79aa was first truncated into three segments: L1 (80–233 aa), L2 (213–368 aa), and L3 (348–526 aa). Expression of the three truncated fusion proteins was confirmed using an anti-GST antibody. Western blotting revealed that 3A1, 3C8, and 6B1 recognized the L2 fragment, 4G9 and 6C9 recognise the L1 fragment, Additionally, none of the mAbs recognised the L3 fragment (Fig.4A). To further characterize the epitopes recognized by these five mAbs, two additional rounds of truncation were performed based on the L1 and L2 fragments, and their reactivity was verified by western blotting (Fig.4B and C). Here, we identified four epitopes recognized by five mAbs through fine mapping: 4G9 recognized the sequence ^144^DEKQFDILNK^153^, 6C9 recognized ^154^AVTSYNKILT^163^, 3A1 and 3C8 recognized ^207^VERDGMQEEA^216^, and 6B1 recognized ^312^ENHKELMHIK^321^ (Fig.5).

### 3.4 Homology and cross-reactivity analysis

**Fig. 6:**
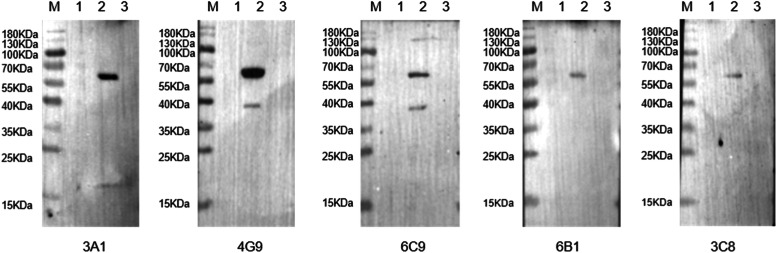
Cross-reactivity assays between mAbs and AHSV and EHDV VP5 proteins. Reactions of mAbs 3A1, 4G9, 6C9, 6B1, and 3C8 with VP5 proteins of three Orbiviruses. Lane 1: rec-EHDV VP5; Lane 2: BTV-1-infected BHK-21 cells; Lane 3: rec-AHSV VP5.

To evaluate the conservation of *Orbivirus* epitopes, the VP5 amino acid sequences of BTV, AHSV, and EHDV were retrieved from GenBank and analyzed using BioEdit software. ^144^DEKQFDILNK^153^ was not conserved across the typical serotypes of BTV (except for BTV-23); ^154^AVTSYNKILT^163^ was not conserved across the typical serotypes of BTV; ^207^VERDGMQEEA^216^ was moderately conserved across the typical serotypes of BTV (except for BTV-3, 5, 6, 8, 9, 13, 14, 18, 20, and 23); and ^312^ENHKELMHIK^321^ was no`t conserved across the typical serotypes of BTV (except for BTV-2, 23). The four epitopes were not conserved in AHSV and EHDV (Fig.5). Cross-reactivity analysis revealed that mAbs 3A1, 4G9, 6C9, 3C8 and 6B1 specifically recognized the VP5 protein of BTV-1 (Fig.6).

### 3.5 Bioinformatics analysis of VP5Δ1–79aa

**Fig. 7:**
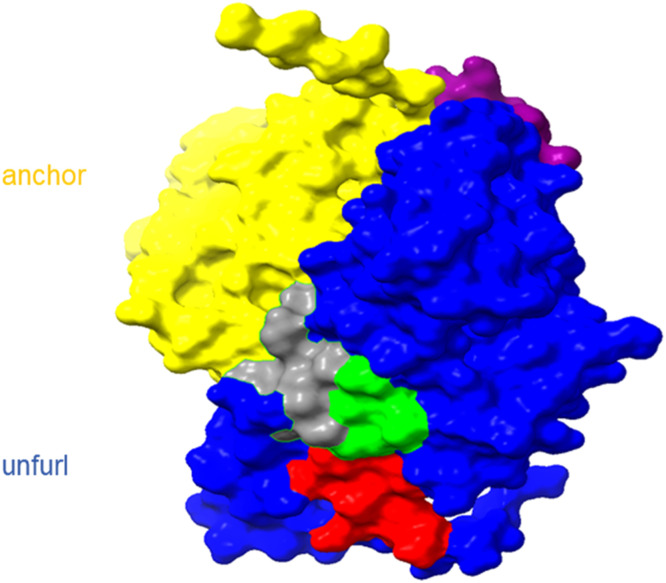
Structural analysis of VP5Δ1–79aa. VP5 structure and five epitopes recognized by mAbs. Yellow and blue represent the monomer structure of VP5. Red represents the sequence ^144^DEKQFDILNK^153^, green the sequence ^154^AVTSYNKILT^163^, purple the sequence ^207^VERDGMQEEA^216^, and gray the sequence ^312^ENHKELMHIK^321^.

The 3D structural of BTV-1 VP5, AHSV-1 VP5, and EHDV-1 VP5 were modeled using SWISS-MODEL (https://swissmodel.expasy.org/) with the 3J9E template. Structural analysis revealed that the epitopes for 4G9,6C9 and 6B1 were situated at the base of the unfurling domain, whereas the 3A1 and 3C8 epitope was located at the top of the unfurling domain (Fig.7).

## 4 Discussion

Although both VP2 and VP5 are potential targets for neutralizing antibodies, current evidence indicates that all known neutralizing epitopes are located on VP2 (Du et al., 2014; Huismans and Erasmus, 1981). VP5 exhibits higher conservation compared to VP2, although it still demonstrates a degree of variability both within and between viruses (Kochinger et al., 2014). However, in other studies, vaccination with VP2 and VP5 or co-expressed proteins (VP2+VP5) enhanced the protective immune response, whereas VP2 alone was ineffective (Ma et al., 2012). Additionally, it has been observed that the amount of VP2 in a vaccine dose can be reduced by more than half when administered with VP5, while still maintaining the same level of protection (Noad and Roy, 2009; Roy et al., 1990).

Epitope-based vaccines can be recognized by both B-cell and T-cell receptors, thereby inducing immune responses. Identification of antigenic epitopes capable of inducing robust immune responses is crucial for the design of epitope-based vaccines (Mortazavi et al., 2024). VP2 and VP5 have been identified as potential key components for BTV subunit vaccines (Stewart et al., 2012; Thuenemann et al., 2013). The smaller outer capsid protein VP5 stimulates the neutralization response, possibly through interactions with VP2 in the viral capsid (Cowley and Gorman, 1987; Mertens et al., 1987). The structural flexibility of VP5, a component of the viral outer capsid, facilitates mutations that allow the virus to evade host antibody responses (Russell et al., 2018). Our study identified epitopes on the VP5 protein that could facilitate its structural elucidation and contribute to preventing immune escape. Our newly identified epitopes exhibit unique sequence characteristics compared to previously reported findings and are located in distinct structural domains. This study not only prompts the utility of these mAbs in BTV-1 serological differential diagnostics but also establishes a foundation for novel BTV-1 vaccine design by delineating four antigenic epitopes on the VP5 protein.

Previous studies predicted five potential epitopes on BTV1 VP5, but these require further experimental validation(Russell et al., 2018). In contrast, predicted and experimentally identified B-cell epitopes on BTV4 VP5 showed no overlap with monoclonal antibody (mAb)-defined epitopes, suggesting that the bioinformatic methods used for epitope prediction may need re-evaluation (Sun et al., 2014). Additionally, BTV16 VP5 was reported to harbor three B-cell epitopes: ³¹⁰ITANTREIQHIKEE³²³, ²⁶⁵LSGID²⁶⁹, and ¹⁸⁸STMVKEYRQKIDALKA²⁰³(Wang et al., 2013). Our experimentally mapped epitopes on BTV-1 VP5 (¹⁴⁴DEKQFDILNK¹⁵³, ¹⁵⁴AVTSYNKILT¹⁶³, ²⁰⁷VERDGMQEEA²¹⁶, ³¹²ENHKELMHI K³²¹) showd no overlap with the predicted epitopes in BTV-1 or the experimentally defined epitopes in BTV-16. This discrepancy with bioinformatic predictions aligns with findings in BTV-4 and BTV-1 and underscores the current limitations of epitope prediction algorithms. More importantly, the complete lack of conservation with BTV-16 epitopes highlights the strong serotype-specificity of VP5.

This study provides the first set of experimentally validated linear B-cell epitopes for BTV-1 VP5. The identification of these nonconserved epitopes establishes a critical foundation for developing BTV-1-specific diagnostic tools and subunit vaccines, addressing a key gap in bluetongue virus.

## 5 Conclusions

In summary, this study generated five mAbs and identified four linear B-cell antigenic epitopes on BTV1 VP5, which is the first time that these epitopes have been screened. Our findings could contribute to the development of BTV1 diagnostic methods, BTV-specific diagnostic assays, epitope-based marker vaccines and provide a foundation for further understanding of the structure and function of the BTV1 VP5 protein.

## Funding

This study was financially supported by the 10.13039/501100012166National Key Research and Development Program of China (2021YFD1800500 and 2024YFD1800100); Science Fund for Creative Research Groups of Gansu Province (22JR5RA024); NBCITS (CARS-37) and ASTIP (CAAS-ASTIP-2021-LVRI).

## Data availability

The datasets and materials from this study can be available upon request to interested researchers.

## Declarations

**Ethics approval** All animals were handled in strict accordance with good animal practice according to the Animal Ethics Procedures and Guidelines of the People's Republic of China, and the study was approved by The Animal Administration and Ethics Committee of Lanzhou Veterinary Research Institute, Chinese Academy of Agricultural Sciences (Permit No LVRIAEC-2024–093).

## Competing interest

The authors declare no competing interests.

## CRediT authorship contribution statement

**Fanhua Meng:** Writing – original draft, Validation, Investigation, Formal analysis, Data curation. **Xuechun Liu:** Validation, Investigation. **Yuqing Song:** Validation, Investigation. **Xinbing Hu:** Validation, Investigation. **Zhancheng Tian:** Resources, Investigation. **Guiquan Guan:** Project administration, Funding acquisition. **Lijie Tang:** Writing – review & editing, Resources. **Hong Yin:** Project administration, Funding acquisition. **Junzheng Du:** Writing – review & editing, Supervision, Resources, Project administration, Methodology, Funding acquisition, Conceptualization.

## Declaration of competing interest

The authors declare that they have no known competing financial interests or personal relationships that could have appeared to influence the work reported in this paper.
